# Transcatheter tricuspid valve replacement with EVOQUE™ in arrhythmogenic right ventricular cardiomyopathy: insights from the first-in-man case report

**DOI:** 10.1093/ehjcr/ytaf684

**Published:** 2026-01-03

**Authors:** Jonas Michael Bodanowitz, Maria Gafiullina, Plamen Kochev, Antonia Ourani, Hüseyin Ince

**Affiliations:** Department of Cardiology, Universitätsmedizin Rostock, Schillingallee 36, 18057 Rostock, Germany; Department of Cardiology, Universitätsmedizin Rostock, Schillingallee 36, 18057 Rostock, Germany; Department of Cardiology, Universitätsmedizin Rostock, Schillingallee 36, 18057 Rostock, Germany; Department of Cardiology, Universitätsmedizin Rostock, Schillingallee 36, 18057 Rostock, Germany; Department of Cardiology, Vivantes Klinikum Am Urban, Dieffenbachstraße 1, 10967 Berlin, Germany; Department of Cardiology, Vivantes Klinikum Neuköln, Rudower Str. 48, 12351 Berlin, Germany; Department of Cardiology, Universitätsmedizin Rostock, Schillingallee 36, 18057 Rostock, Germany; Department of Cardiology, Vivantes Klinikum Am Urban, Dieffenbachstraße 1, 10967 Berlin, Germany; Department of Cardiology, Vivantes Klinikum Neuköln, Rudower Str. 48, 12351 Berlin, Germany

**Keywords:** arrhythmogenic right ventricular cardiomyopathy (ARVC), Severe tricuspid regurgitation, Transcatheter tricuspid valve replacement (TTVR), First-in-man, Right heart failure, Case report

## Abstract

**Background:**

Severe functional tricuspid regurgitation (TR) in the setting of arrhythmogenic right ventricular cardiomyopathy (ARVC) represents a challenging clinical entity, often complicated by progressive right ventricular (RV) dysfunction and limited interventional options.

**Case summary:**

We report the first worldwide case of successful transcatheter tricuspid valve replacement (TTVR) with the EVOQUE™ system in a 37-year-old patient with ARVC, severe TR, and a cardiac resynchronization therapy defibrillator (CRT-D), following failed transcatheter edge-to-edge repair (TEER). The procedure resulted in immediate elimination of TR and the patient experienced marked symptomatic improvement.

**Discussion:**

This case highlights the feasibility of TTVR in complex RV pathology, underscoring procedural considerations such as lead–valve interaction, risk of afterload mismatch, and prevention of right heart failure. TTVR with the EVOQUE™ system is feasible in selected patients with ARVC, severe functional TR, prior failed repair, and existing CRT-D leads. Success depends on meticulous pre-procedural planning, intra-procedural imaging, and vigilant haemodynamic management to mitigate RHF risk. This case broadens the spectrum of structural interventions in patients with ARVC and symptomatic TR not suitable for surgery or TEER and supports consideration of TTVR as a bridge-to-transplant strategy.

Learning points

- Transcatheter tricuspid valve replacement (TTVR) with the EVOQUE™ system can be safely performed in patients with arrhythmogenic right ventricular cardiomyopathy (ARVC).
- Patients with ARVC are prone to acute right heart failure after TTVR due to limited RV reserve; proactive haemodynamic optimization can prevent afterload mismatch.
- TTVR may serve as a bridge-to-transplant strategy in selected ARVC patients with severe tricuspid regurgitation after failed transcatheter edge-to-edge repair.

## Introduction

Arrhythmogenic right ventricular cardiomyopathy (ARVC) encompasses a spectrum of inherited disorders characterized by fibrofatty replacement of the right ventricular myocardium, with a predominant arrhythmic presentation. First described in the late 1970s and early 1980s, ARVC is now recognized to often involve both ventricles, with an estimated prevalence of 0.078% and a slight male predominance.^1,2^ Functional TR secondary to RV dilation is common in ARVC due to progressive fibrofatty myocardial replacement and annular dilatation.^3^ Severe TR worsens prognosis by exacerbating systemic congestion, impairing forward output, and accelerating RV dysfunction.^4^ Management is challenging, surgical risk is often prohibitive, and percutaneous repair devices may be ineffective in advanced disease with massive annular dilation and leaflet tethering.^5^

Alongside edge-to-edge repair, new transcatheter approaches have been developed, including the EVOQUE™ TTVR system, which was recently evaluated in the TRISCEND II trial and demonstrated high procedural success in functional TR.^6^

We present the first reported case of EVOQUE™ implantation in ARVC with severe TR after failed TEER therapy, emphasizing technical strategies and management of potential RV failure.

## Summary figure

**Figure:**
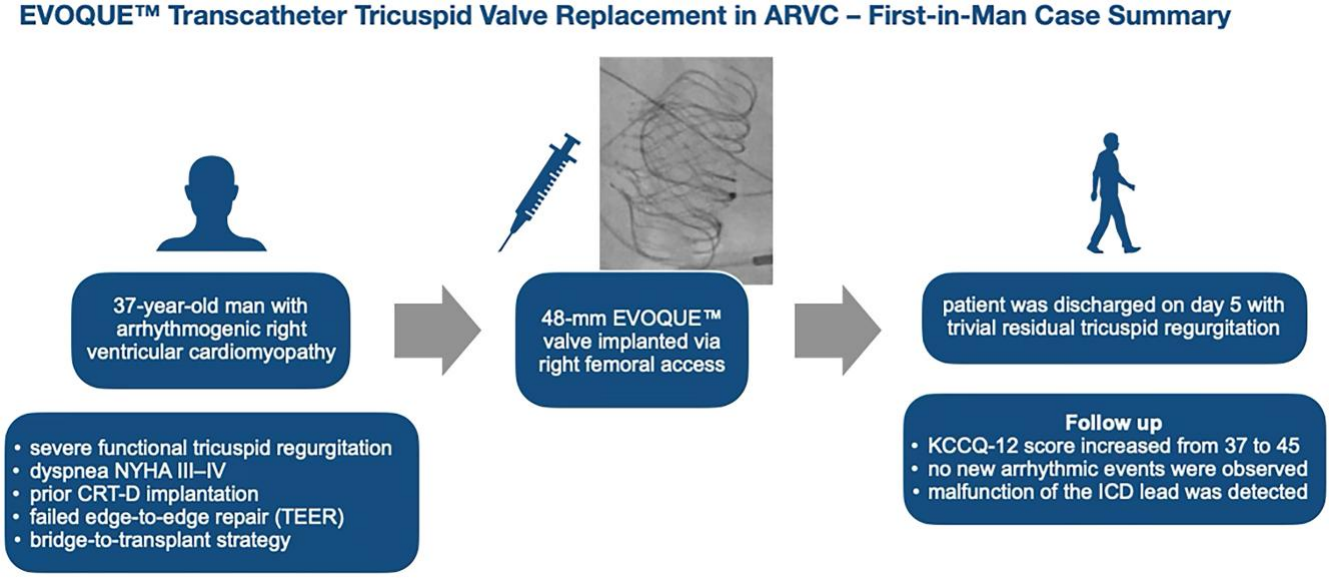


## Case presentation

A 37-year-old man with genetically confirmed arrhythmogenic right ventricular cardiomyopathy (ARVC) was electively admitted for evaluation of further therapeutic options after a failed transcatheter tricuspid edge-to-edge repair (TEER) in the setting of severe, symptomatic tricuspid regurgitation (TR). He presented with progressive decline in exercise capacity, dyspnoea classified as NYHA III–IV, and peripheral oedema. His clinical course was characterized by escalating diuretic requirements and persistent severe TR, and he was concurrently evaluated for heart transplantation.

During the preceding 2 years, he had been treated with flecainide and sotalol without major arrhythmic events. At admission, medication consisted only of oral torasemide. Due to progressive atrioventricular conduction disease, a cardiac resynchronization therapy defibrillator (CRT-D) had been implanted in 2013. He had also undergone both endocardial and epicardial ablations for ventricular tachycardia in 2015, 2018, and 2019. Prior right and left heart catheterization excluded coronary artery disease and pulmonary hypertension.

**Figure 1:**
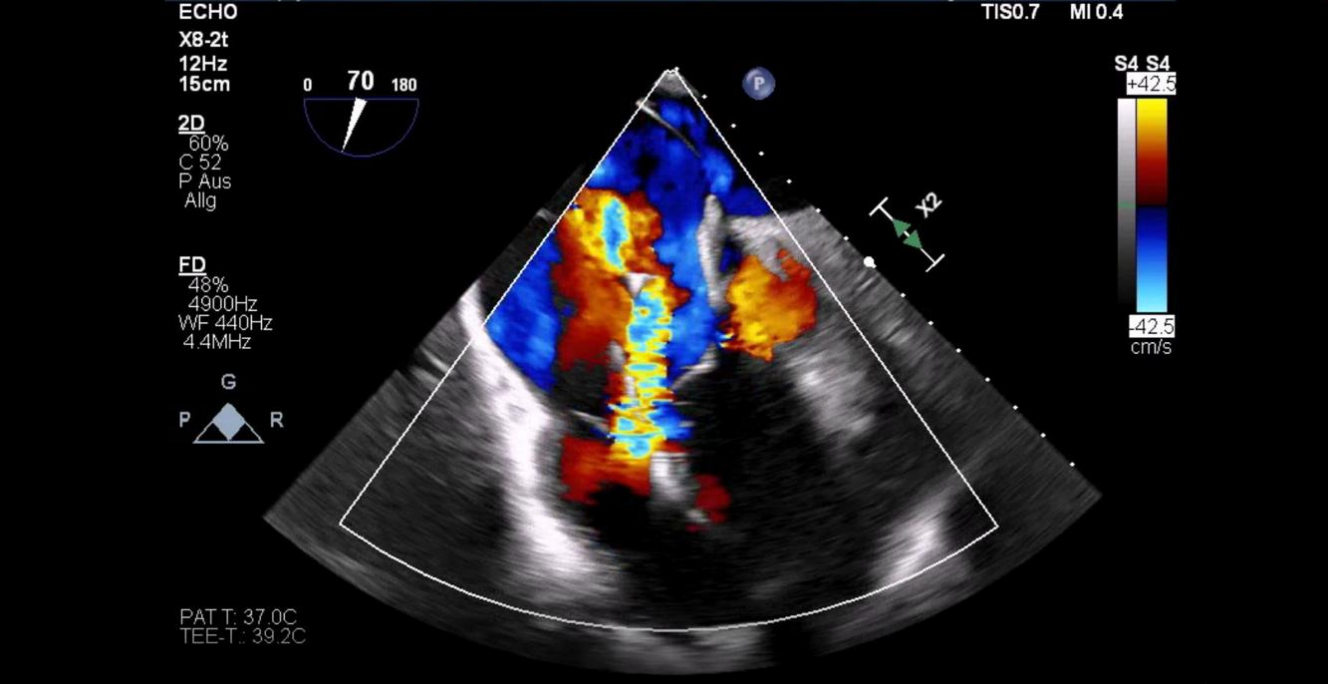
Transoesophageal echocardiography demonstrating severe tricuspid regurgitation prior to EVOQUE™ implantation. The regurgitant jet is aligned with the intracardiac device lead.

Transthoracic and transoesophageal echocardiography showed a normal left ventricle with preserved systolic function, without hypertrophy or regional wall motion abnormalities. Findings were consistent with ARVC, including paradoxical septal motion and hypokinesia to akinesia of the RV free wall. The RV was severely dilated with impaired systolic function (RVEDD 70 mm, TAPSE 16 mm). The right atrium was moderately dilated, and severe TR (vena contracta 22 mm) was observed along the pacemaker lead (*Figure 1*). The mitral and aortic valves were competent, and the ascending aorta appeared normal. Laboratory testing was unremarkable except for elevated NT-proBNP at 950 pg/mL.

**Figure 2:**
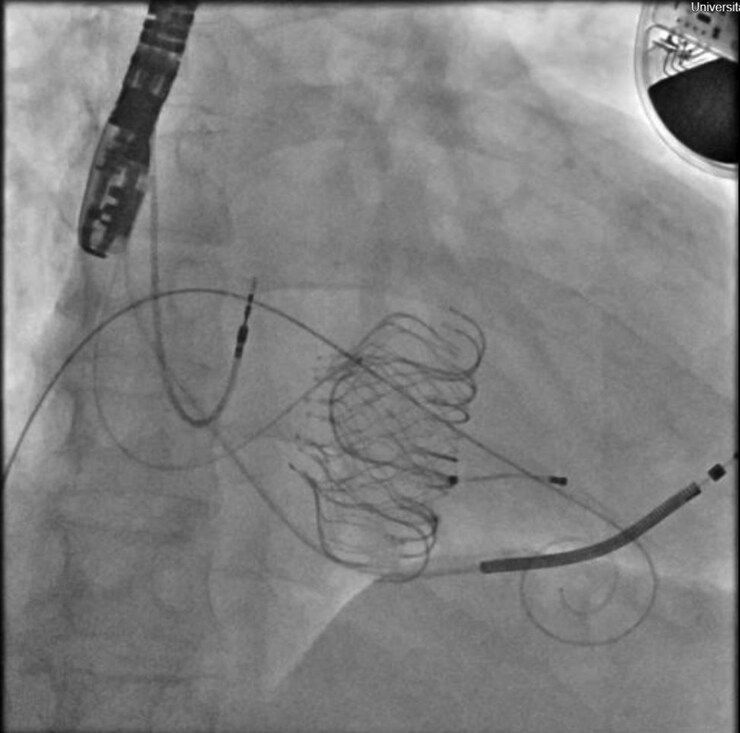
Intraprocedural fluoroscopic image during EVOQUE™ tricuspid valve implantation.

Given prohibitive surgical risk and failed TEER at another institution, the Heart Team recommended transfemoral transcatheter tricuspid valve replacement (TTVR) with the EVOQUE™ system as a bridge-to-transplant strategy. Under general anaesthesia with fluoroscopic and three-dimensional transoesophageal guidance, a 48 mm EVOQUE™ valve was advanced via the right femoral vein and deployed with excellent coaxial alignment and secure anchoring (*Figure 2*). Care was taken to avoid CRT-D lead entrapment; fluoroscopy and device interrogation confirmed preserved lead stability and function.

**Figure 3:**
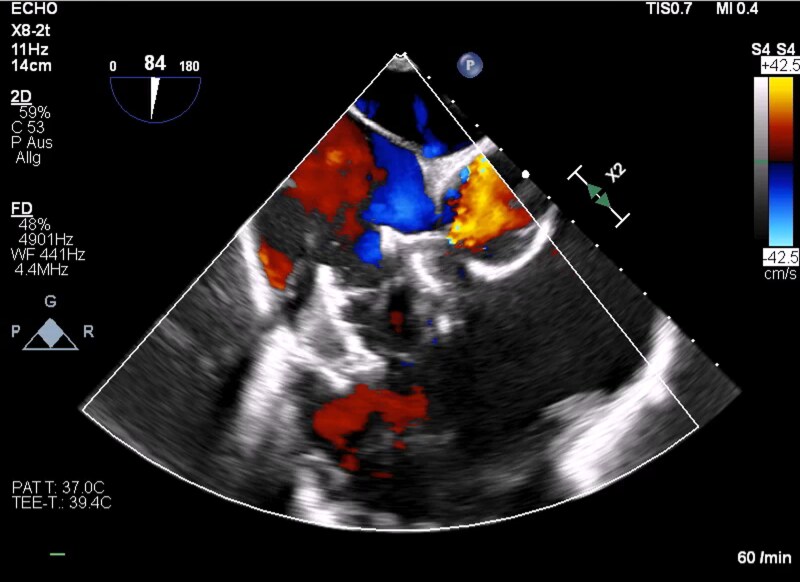
Transoesophageal echocardiography after EVOQUE™ implantation showing markedly reduced tricuspid regurgitation with trivial residual flow.

Post-procedural monitoring included temporary norepinephrine support for haemodynamic stabilization, which was quickly discontinued. No arrhythmic events occurred. Echocardiography showed trivial residual TR (grade 0–I) with slight improvement in RV function (*Figure 3*). CRT-D interrogation confirmed preserved device performance. The postoperative course was uneventful, and the patient was discharged on Day 5 in NYHA class II without diuretic therapy. The patient was discharged in stable condition on apixaban 5 mg 1-0-1 for anticoagulation, in line with contemporary post-TTVR management practice.

At follow-up, quality of life improved, with KCCQ-12 score increasing from 37 to 45. Two weeks later, an ICD lead malfunction was detected with high shock impedance (>750 ohms). No new arrhythmic events or device-related rhythm disturbances were observed following EVOQUE™ implantation. Lead extraction was not feasible due to fixation through the prosthesis. Given his pacemaker dependency, subcutaneous or extravascular ICDs were not viable. Options discussed included epicardial shock coil implantation or use of a coronary venous lead, but these were deemed unsuitable. Together with the patient, we opted for implantation of a very thin OmniaSecure ICD lead through the EVOQUE™ valve. Approval for humanitarian use has been requested, and in the interim the patient is protected with a wearable cardioverter defibrillator (WCD).

## Discussion

The coexistence of arrhythmogenic right ventricular cardiomyopathy (ARVC), severe tricuspid regurgitation (TR), and an implanted CRT-D system presents unique diagnostic and therapeutic challenges. ARVC leads to progressive RV dilation, impaired contractility, and arrhythmias; functional TR develops due to annular enlargement and leaflet tethering, further worsening right heart failure and prognosis.^3,4^ Transcatheter edge-to-edge repair (TEER) has emerged as a treatment option for functional TR but is limited in cases with massive annular dilation, large coaptation gaps, and marked leaflet tethering.^5^ Our patient exemplified these limitations, as prior TEER failed to achieve durable TR reduction. This supports valve replacement strategies in anatomically complex cases.

The EVOQUE™ TTVR system, validated in the TRISCEND II trial, has demonstrated high procedural success and symptomatic improvement in patients with functional TR.^6^ To date, there are no published experiences of EVOQUE™ implantation in ARVC. This case therefore represents the first report, extending the applicability of TTVR to a population with complex RV pathology, where it may serve as a bridge-to-transplant strategy.

Several ARVC-specific risks must be considered. First, fibrofatty myocardial replacement increases the risk of RV perforation during wire and valve manipulation, necessitating meticulous technique. Second, pro-arrhythmic potential during instrumentation exists, although no ventricular tachycardia occurred in this case. Third, device leads may interfere with valve positioning or function.^7^ Although implantation was acutely successful, an ICD lead malfunction developed during follow-up, underlining the importance of long-term monitoring and individualized lead–valve management strategies. Fourth, patients with ARVC are especially prone to right heart failure (RHF) due to limited RV reserve. Sudden elimination of severe TR can abruptly increase afterload (‘afterload mismatch’).^8,9^ Preventive measures include preload optimization, cautious fluid balance, judicious inotrope use, and availability of mechanical support.^10,11^ In our case, early stabilization with norepinephrine and careful postoperative management likely contributed to the absence of RHF despite advanced RV disease.

From a clinical perspective, three points emerge: (i) TTVR can be performed successfully in patients with ARVC and advanced RV remodelling when surgery is prohibitive and TEER has failed; (ii) safe integration with pre-existing CRT-D systems is feasible but requires close follow-up; (iii) TTVR may serve as a bridge-to-transplant, improving symptoms and stability until definitive therapy is possible.

Uncertainties remain regarding long-term durability, progressive RV remodelling and risk of valve dysfunction in device-bearing patients. Registry data and longer follow-up are needed to assess whether TTVR alters the natural history of ARVC or primarily provides symptomatic relief.

## Conclusion

TTVR with the EVOQUE™ system is feasible in selected patients with ARVC, severe functional TR, prior failed repair, and existing CRT-D leads. Success depends on meticulous pre-procedural planning, intra-procedural imaging, and vigilant haemodynamic management to mitigate RHF risk. This case broadens the spectrum of structural interventions in patients with ARVC and symptomatic TR not suitable for surgery or TEER. TTVR may serve as a bridge-to-transplant strategy for patients with ARVC under these clinical scenarios.

## Lead author biography


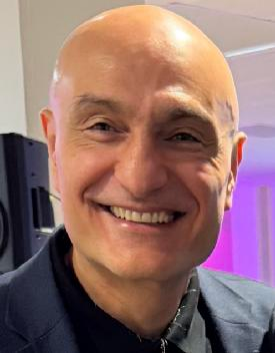


Hüseyin Ince is a Professor of Cardiology (since 2008). In October 2013, he serves as Director of the Department of Cardiology at Vivantes Klinikum Am Urban and Klinikum im Friedrichshain in Berlin. Since April 2017, he serves as Director of the Clinic of Cardiology at the University of Rostock. In addition, he serves as Director of the Department of Cardiology at Vivantes Klinikum Am Urban and, since 2022, also at Vivantes Klinikum Neukölln in Berlin.

## Author contributions

Jonas Michael Bodanowitz (Conceptualization [supporting], Data curation [supporting], Writing—original draft [equal], Writing—review & editing [supporting]), Maria Gafiullina (Conceptualization [supporting], Writing—original draft [supporting], Writing—review & editing [supporting]), Plamen Kochev (Conceptualization [supporting], Writing—original draft [supporting], Writing—review & editing [supporting]), Antonia Ourani (Conceptualization [supporting], Visualization [supporting], Writing—original draft [supporting], Writing—review & editing [supporting]), and Hüseyin Ince (Conceptualization [supporting], Data curation [supporting], Writing—original draft [equal], Writing—review & editing [supporting])

**Consent:** The authors confirm that written consent for submission and publication of this case report including images and associated text has been received from the patient in line with the Committee on Publication Ethics (COPE) guidelines.

## Data Availability

The data underlying this article will be shared on reasonable request to the corresponding author.
